# S212. CONVERSING WITH PEOPLE WITH THOUGHT DISORDER

**DOI:** 10.1093/schbul/sby018.999

**Published:** 2018-04-01

**Authors:** Cherrie Galletly, David Ash, Shaun Sweeney, Jonathan Crichton

**Affiliations:** 1 The University of Adelaide; 2 The Adelaide Clinic; 3 Northern Adelaide Local Health Network; 4 University of South Australia

## Abstract

**Background:**

Thought disorder is a common symptom in psychotic disorders such as schizophrenia. In the research and training literature, thought disorder is assumed to prevent any useful conversation (Galletly & Crichton, 2011). It is depicted as something to be sampled and analysed, rather than as a factor that modifies, but does not prevent, meaningful communication.

Psychiatrists routinely interact with their thought disordered patients as part of day-to-day clinical care. The study investigates the skills and strategies used by psychiatrists in clinical interviews with patients with thought disorder. The importance of this study is that identification and detailed description of these specific interview techniques will enable them to be used in the training of psychiatrists and other mental health clinicians.

**Methods:**

Twenty-four routine interviews between inpatients with thought disorder and their treating psychiatrists were recorded and transcribed. All participants gave written informed consent and this study was approved by the institutional ethics committees.

The transcripts were examined by the research team (an applied linguist, two psychiatrists and mental health social worker). Excerpts were subsequently presented at two workshops, attended by psychiatrists, trainee psychiatrists and junior medical staff. Participants were asked to identify and describe the techniques used by the psychiatrists in the course of their interviews with thought disordered patients.

**Results:**

The interviews were generally quite brief (mean duration 19.24 (SD 7) minutes), and had many characteristics in common. The tone was conversational, with a normal turn-taking structure, few repairs and an easy flow despite the often-disjointed content. The psychiatrists were not confrontational or judgemental. During the first half of the interview, there was often a period of delusional, thought disordered discourse, in which psychiatrists engaged through close attention to the patient’s language, navigating this while commenting and sometimes asking questions. The purpose seemed to be to build rapport by ensuring the patient felt they had been listened to, and this period of relatively uninterrupted speech provided the psychiatrist with the opportunity to assess the patient’s mental state. Rather than comment on the content, psychiatrists often engaged with the feelings (e.g. ‘stressed”) that arose in response to the psychotic experiences described by patients.

It was clear that both parties contributed to the agenda for the conversation. The patients were active participants, often with specific questions or concerns, and these were answered carefully and respectfully regardless of content. Psychiatrists observed that they were also powerless about some matters of particular concern to patients (e.g. the no-smoking policy). Discharge planning was a substantial component of the interviews, as patients wished to leave hospital as soon as possible, and psychiatrists have institutional pressures to reduce the length of stay. The psychiatrists generally assessed insight, but did not undertake formal risk assessments. They provided explanations of the patient’s legal status, current treatments, and planned treatments (such as ECT).

**Discussion:**

Thought disorder does not exist in isolation, as a phenomenon to be sampled; rather it modifies but does not prevent meaningful conversation and exchange of ideas and information. Experienced psychiatrists are able to undertake meaningful, useful interviews with people with thought disorder. The skills involved have not been described previously. These findings h can be used to develop training resources for mental health clinicians who will be working with people with psychotic disorders.

